# Saphenous Vein Sparing Superficial Inguinal Dissection in Lower Extremity Melanoma

**DOI:** 10.1155/2014/652123

**Published:** 2014-07-13

**Authors:** Muhammed Beşir Öztürk, Arzu Akan, Özay Özkaya, Onur Egemen, Ali Rıza Öreroğlu, Turgut Kayadibi, Mithat Akan

**Affiliations:** ^1^Department of Plastic Reconstructive and Aesthetic Surgery, Tekirdag Government Hospital, 59020 Tekirdag, Turkey; ^2^Department of General Surgery, Okmeydani Training and Research Hospital, 34445 Istanbul, Turkey; ^3^Department of Plastic Reconstructive and Aesthetic Surgery, Okmeydani Training and Research Hospital, 34445 Istanbul, Turkey; ^4^Department of Plastic Reconstructive and Aesthetic Surgery, Prof. Dr. A. Ilhan Ozdemir State Hospital, 28000 Giresun, Turkey; ^5^Department of Plastic Reconstructive and Aesthetic Surgery, Medipol University Hospital, 34200 Istanbul, Turkey

## Abstract

*Aim*. The classic inguinal lymph node dissection is the main step for the regional control of the lower extremity melanoma, but this surgical procedure is associated with significant postoperative morbidity. The permanent lymphedema is the most devastating long-term complication leading to a significant decrease in the patient's quality of life. In this study we present our experience with modified, saphenous vein sparing, inguinal lymph node dissections for patients with melanoma of the lower extremity. *Methods*. Twenty one patients (10 women, 11 men) who underwent saphenous vein sparing superficial inguinal lymph node dissection for the melanoma of lower extremity were included in this study. The effects of saphenous vein sparing on postoperative complications were evaluated. *Results*. We have observed the decreased rate of long-term lymphedema in patients undergoing inguinal lymphadenectomy for the lower extremity melanoma. *Conclusion*. The inguinal lymphadenectomy with saphenous vein preservation in lower extremity melanoma patients seems to be an oncologically safe procedure and it may offer reduced long-term morbidity.

## 1. Introduction

Regional lymph node dissection is the standard treatment regimen for patients with sentinel lymph node biopsy (SLNB) positive melanoma or clinically evident palpable lymph node metastasis of the disease. Inguinal lymph node dissection is the main step for the regional control of the lower extremity melanoma, but this surgical procedure is associated with significant postoperative morbidity. Wound complication rates up to 71% have been reported, including hematoma, seroma, skin necrosis, wound infection, and wound dehiscence [1]. The permanent lymphedema is the most devastating long-term complication leading to a significant decrease in the patient's quality of life [2].

Many techniques have been reported to reduce postoperative lymphedema, such as preserving the muscle fascia [3], pedicled omentoplasty [4], sartorius transposition [5], and saphenous vein sparing inguinal lymphadenectomy [6]. The reported studies on sparing the saphenous vein in inguinal node dissection suggest a reduced rate of lymphedema and other postoperative complications [6, 7]. Randomized controlled trials are needed to prove the benefits of various technical modifications.

The classic inguinal lymphadenectomy includes en bloc removal of all lymph node bearing fibrofatty tissue and the saphenous vein within the femoral triangle. Catalona defined the saphenous vein sparing inguinal lymphadenectomy, postulating a decrease in the postoperative complication rates in vulvar and penile malignancies [6].

In this study, we present our experience with sparing the saphenous vein during inguinal lymph node dissections for patients with melanoma of the lower extremity. The effects of saphenous vein sparing on postoperative complications were evaluated.

## 2. Patients and Methods

Twenty-one patients (10 women, 11 men) who underwent saphenous vein sparing superficial inguinal lymph node dissection for the melanoma of lower extremity between February 2011 and April 2013 were included in this study.

Melanoma diagnoses were based on pathologic investigations and all patients were histologically diagnosed prior to surgery. All patients were staged clinically.

Lymph node dissection was performed on patients with clinically detectable inguinal lymph node metastases, for SLNB positive patients and for patients with thick (>4 mm) primary melanomas.

**Figure 1:**
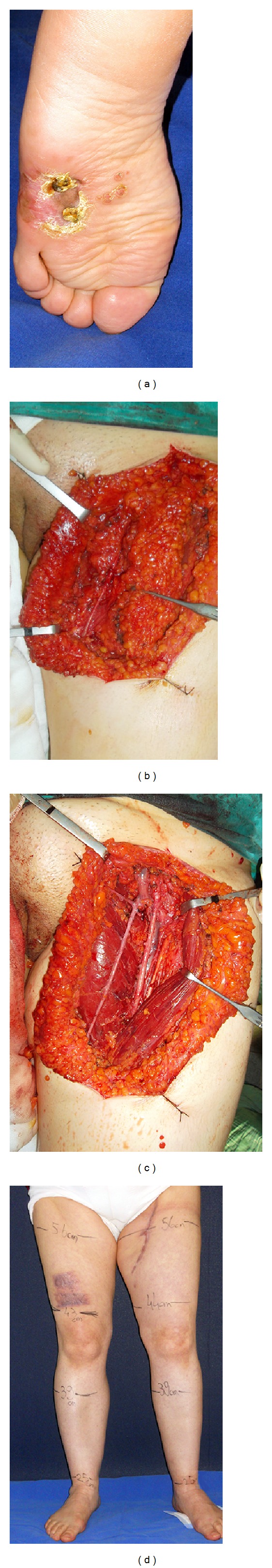
(a) 45-year-old female patient having T4 (4 mm) melanoma of the left plantar foot. (b) Intraoperative view of the sparing long saphenous vein. (c) En block removal of lymph node bearing fibrofatty tissue. (d) The 12th month follow-up of the patient without any sign of lymphedema.

Inguinal lymph node dissection was performed through a standard 12 cm incision extending from 2 cm below the inguinal ligament to the apex of the femoral triangle. All the fibrofatty tissue, extending from the external oblique aponeurosis 2 cm above the inguinal ligament to the medial border of the adductor longus muscle medially and sartorius muscle laterally, was removed. According to the saphenous vein preserving inguinal lymph node dissection technique described by Catalona, the main truncus of the saphenous vein was found at the level of femoral artery entry point and was preserved during the dissection (Figure 1) [6].

After completion of the dissection, all the vascular compromised skin was excised. Suction drains were used routinely. All the patients were administered with low molecular weight heparin 6 hours postoperatively for deep vein thrombosis prophylaxis and prophylactic antibiotics. The patients were observed for any short-term complications and were discharged when the suction drainage was less than 40 cc in 24 hours.

All patients were called for regular visits at the postoperative 1st week, postoperative 2nd week, postoperative 6th week, postoperative 6th month, postoperative 1st year, and postoperative 18th month at the outpatient clinic. Patients were asked to wear compressive garments for 3–6 months during the postoperative period. The day before the visit they were asked to take off the compressive garments.

Patient's demographic characteristics and associated comorbidities were analyzed. During observations, prospective assessment of the wound complications including wound dehiscence, skin necrosis, wound infection, seroma, and hematoma as well as palpable inguinal lymph nodes and locoregional recurrences was noted. Pathologic information included Breslow thickness, ulceration of the primary tumor, total number of the excised nodes, and the number of the positive nodes.

A short-term complication was defined as an occurrence within the first 6 months of the operation and a long-term complication was any complication occurring after that period.

Wound infection was defined by the use of antibiotics for culture-proven infected drainage postoperatively and wound dehiscence was described as wound healing problem with a measured defect of at least 1 cm in length. Seroma was defined as a palpable subcutaneous fluid collection at the operation area requiring percutaneous drainage.

**Figure 2:**
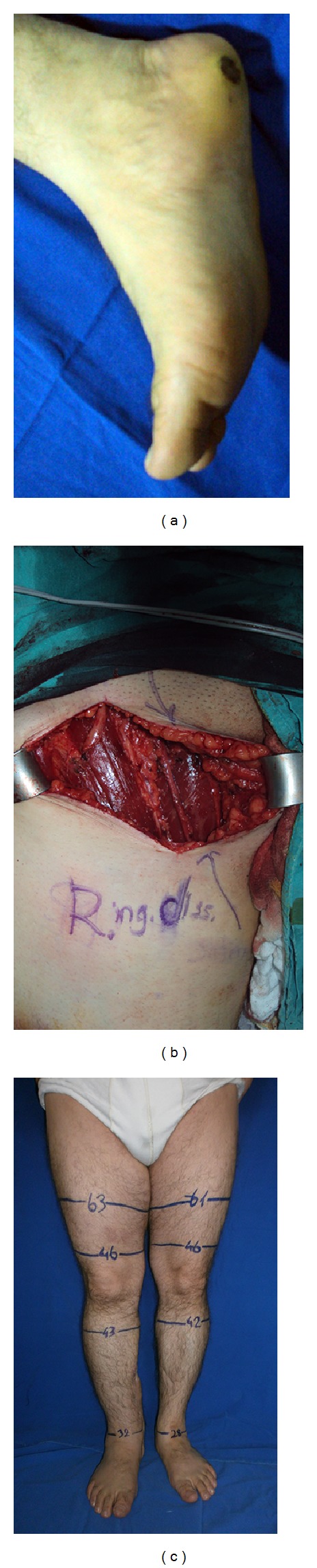
(a) Preoperative view of the 43-year-old male patient having T4 (4,8 mm) melanoma located to the right heel. (b) Right-sided saphenous vein superficial inguinal dissection, intraoperative view. (c) Postoperative 16th month picture, showing no sign of lymphedema (note that the right ankle is thick because of the use of posterior tibial artery perforator flap in the right heel reconstruction).

Lymphedema was determined as a change equal to or greater than 7% of the sum of all the circumferences (of the predetermined 4 circumference measurement points) between the two legs [8]. Patients were followed up for the development of lymphedema and limb circumference measurements were performed for both legs preoperatively and during the regular visits. Measurements were done at the points of the medial malleolus, 10 centimeters below the medial tibial condyle (MTC), 10 centimeters above the MTC, and the midpoint between anterior superior iliac spine and MTC (Figure 2).

## 3. Results

Twenty-one patients (10 women, 11 men) were included in this study. The median age at diagnosis of the melanoma was 48 years (range 39–68 years).

The average Breslow depth of the primary melanomas was 4,2 mm (1,2 mm–8 mm). Five patients underwent inguinal dissection after groin lymphadenopathy was noted on physical examination at the time of primary lower extremity melanoma diagnosis, 3 patients had positive SLNB, one patient had wide spread in-transit metastasis, and the other 12 patients underwent dissection for primary thick melanomas (≥4 mm).

The mean follow-up period was 14.8 months. The follow-up period was 18 months for 12 patients, 12 months for 7 patients, and 6 months for 2 patients.

Twenty patients did not show any local or regional recurrences or systemic metastasis during the follow-up period. Only one patient (who had widespread in-transit metastasis at the first admission) developed pulmonary metastasis 6 months after the operation and he was lost during the follow-up.

Five short-term complications (23.8%) were observed related to the inguinal area. Seroma formation was noted in 3 patients (14.3%) and hematoma formation was noted in 2 patients (9.5%). There was no noted occurrence of wound infection or wound dehiscence.

Short-term lymphedema formation was observed in 3 patients (3/21, 14.2%) at the 2nd week, in 8 patients (8/21, 38%) at the 6th week, and in 6 patients (6/21, 28,5%) at the 6th month. Long-term lymphedema was noted in 2 patients (2/19, 10.5%) at the 12th month. There was not any persistent lymphedema formation at the 18th month follow-up of 12 patients (0/12).

## 4. Discussion

Inguinal lymph node dissection is associated with significant morbidity despite the refinements in surgical techniques. Complications with inguinal dissections are significantly more common than the other regional lymph node dissections and tend to be a rule rather than exception [1].

Complications following inguinal dissection can be classified into short-term/wound complications and long-term/lymphedema formation. The most frequent wound complications are wound infections, wound dehiscence, seroma formation, and hematoma formation. Serpell et al. reported an overall incidence of wound complication rate as high as 71% after inguinal lymph node dissection for melanoma with a 25% incidence of infection, 25% incidence of delayed wound healing, 46% incidence of seroma, and 29% incidence of lymphedema [1]. Similarly, Chang et al. found 77% wound complication rate after inguinal dissection for melanoma in a prospective study with 55% incidence of infection, 53% incidence of wound dehiscence, 28% incidence of seroma, and 45% incidence of lymphedema [9].

Wound infections/necrosis were frequently observed after inguinal dissections with the prevalence rates 7–55% reported in the literature [1, 9–11]. The wide discrepancies in the incidence of reported complication rates may be attributed to the retrospective design of the studies [8]. Also, there is no universally accepted description of these complications. In our study, wound infection was defined by the use of antibiotics for culture-proven infected drainage postoperatively and wound dehiscence was described as wound healing problem with a measured defect of at least 1 cm. Wound infections/necrosis were not observed in our prospective study [9]. These clear definitions for the complications in our study contribute the low reported incidence. A second explanation for the low incidence of complications is that our patients were relatively young (mean 48 years) with minimal associated comorbidities. Aseptic surgical technique and removal of the vascular compromised skin during the procedure may further help the low complication rate.

Seroma formation was observed in 3 patients (14.3%) and hematoma formation was noted in 2 patients (9.5%) in our study. Seromas were managed with sterile aspirations in outpatient clinic and no additional treatment was needed for these patients. Studies show that the incidence of hematoma/seroma formation ranges from 2 percent to 42 percent and our complication rates were similar to the literature [10–15].

The most debilitating long-term morbidity after inguinal dissection is chronic lymphedema. Lymphedema is a progressive pathological condition in which there is an accumulation of a protein rich fluid and subsequent inflammation, adipose tissue hypertrophy, and fibrosis. Physicosocial morbidity, decreased extremity function, cellulitis, epidermal lymph leak (lymphorrhea), and lymphangiosarcoma development are observed in lymphedema patients. These conditions further diminish the patients' quality of life [16].

The reported incidence of lymphedema after inguinal dissection for melanoma varies widely, ranging from 9 percent to 64 percent [1, 10–14, 16]. Wide range of reported difference is related to the problem that there is no universally accepted definition of the lymphedema also. Some authors define lymphedema as the patients self-complaint about the presence of lymphedema [1], a greater than 2 cm circumference increase compared to the contralateral limb [17], and a volume difference of more than 20% between limbs [18]. All these definitions have their limitations. For example, the effect of the same volume increase in a small person's limb is more prominent than in a larger person's limb. Also, the use of >2 cm circumference difference for the definition of lymphedema does not comprise the severity of the impairment. Spillane et al. studied the definition of lymphedema and suggested 2 alternative equally appropriate definitions of lymphedema, the whole perometer percentage change ≥15% and the sum of circumferences (of the predetermined measurement points) percentage change ≥7 [8]. The optoelectric perometer is not readily available at our clinic and so we used the sum of circumferences percentage change in this study.

We have noticed that lower limb lymphedema was the worst in the first six months and it gradually improved. This is a previously reported pattern [19], but it was striking that, at the 12th month measurements, the lymphedema incidence was found to be 10.5% and lymphedema disappeared completely after 18 months. Review of the literature reveals high prevalence of long-term lymphedema in inguinal dissections ranging from 9 percent to 64 percent and it can be concluded that the procedure of saphenous vein preserving inguinal dissection has been associated with a lower incidence of lymphedema in lower extremity melanoma patients.

Majority of our patients had primary thick (≥4 mm) melanomas. Melanomas thicker than 4.00 mm have a high risk of systemic disease and approximately 40% of them have clinically unapparent nodal involvement at the time of primary diagnosis [20]. Although sentinel lymph node biopsy has gained wide spread acceptance for its safety and minimal morbidity, it is widely used for the intermediate thickness melanomas (1 mm–4 mm) and its use in thick melanomas (≥4 mm) is unclear [21]. With a follow-up period ranging from 6 to 18 months, no patient experienced local or regional recurrence of the primary lesion. All patients, except for one who developed systemic metastasis and was lost during the follow up, continue to do well with no evidence of recurrence, and they are free from the disease. Although the time period for the follow-up is relatively short and the number of patients is relatively low, this technique seems to be an oncologically safe procedure for lower extremity melanoma patients.

During the past century, the original destructive lymph node dissections have been improved with preserving the nonlymphatic structures to limit the surgery related morbidity [22]. In radical neck dissections, the dissections of the internal and external jugular veins often cause maxillofacial edema due to the poor face venous reflux or cause intracranial hypertension and subsequently dizziness and headache [23]. To prevent such complications, modified radical neck dissection, which preserves important structures, such as the internal jugular vein, sternocleidomastoid muscle, and accessory nerve, was described by Suárez, in 1963 [24]. This technique was refined and popularized by various authors in the literature [22, 25, 26]. Modified or “functional” neck dissection avoids much of the morbidity of radical neck dissection while achieving equivalent degrees of control of regional disease in properly selected cases [22].

Also, in inguinal dissections, saphenous vein sparing dissections were described for vulvar and penile malignancies in the literature to avoid postoperative complications such as lymphedema [13, 15]. It is suggested that saphenous vein sparing is associated with a decreased risk of postoperative morbidity without compromising outcomes [6, 13, 15].

Although the exact mechanism of the preserving of a nonlymphatic tissue, the saphenous vein, and the decreased rate of the lymphedema is not clear, it is suggested that the increased venous reflux and subsequent decreased pressure in the venous end and lymphaticovenous connections within the saphenous vein territory may play a role [23, 27, 28].

In conclusion, the inguinal lymphadenectomy with saphenous vein preservation in lower extremity melanoma patients seems to be an oncologically safe procedure and it may offer reduced long-term morbidity.
